# Gamma glutamyl transferase as a biomarker to predict contrast-induced nephropathy among patients with acute coronary syndrome undergoing coronary interventions: a meta-analysis

**DOI:** 10.1097/MS9.0000000000000967

**Published:** 2023-06-20

**Authors:** Mona Javid, Arian Mirdamadi, Mohammadreza Javid, Ehsan Amini-Salehi, Azin Vakilpour, Mohammad-Hossein Keivanlou, Parham Porteghali, Soheil Hassanipour

**Affiliations:** a Student Research Committee, School of Medicine; b School of Medicine; c Gastrointestinal and Liver Diseases Research Center, Guilan University of Medical Sciences, Rasht, Iran

**Keywords:** acute coronary syndrome, contrast-induced nephropathy, gamma-glutamyl transferase, meta-analysis, systematic review

## Abstract

The third most frequent reason for hospitalized acute kidney injury is contrast-induced nephropathy (CIN). Percutaneous coronary intervention (PCI) and coronary angiography (CAG) are two interventions that can result in CIN. In this study, we sought to determine how well gamma-glutamyl transferase (GGT) can predict CIN following CAG and PCI.

**Method:**

Two researchers searched through PubMed, Scopus, and Web of Science in November 2022 to find articles that examined GGT levels in CIN patients following PCI or CAG. To rate the quality of the studies, the Joanna Briggs Institute Critical Appraisal Checklist was employed. The Cochran test and I^2^ statistics were utilized to assess study heterogeneity. To calculate the number of participants required to reject the null hypothesis, power analysis was used. We evaluated the epidemiologic strength of the results using the Grading of Recommendations Assessment, Development, and Evaluation (GRADE). The authors used Comprehensive Meta-analysis Version 3 to summarize the results.

**Results:**

GGT was shown to be considerably greater in patients with CIN according to the meta-analysis’s findings (odds ratio: 3.21, 95% CI: 1.26–8.15, *P*=0.014); nevertheless, the findings were accompanied by significant heterogeneity (I^2^=91.93%, *P*<0.001). Although the relationship between CIN and GGT was power full regarding power analysis (1- β =1, number of effect sizes=4, the average number per group=336), very low quality of evidence was observed regarding GRADE criteria.

**Conclusions:**

These results suggest the GGT level may be a predictor of contrast-induced nephropathy in patients having cardiac catheterization; however, more research is required to prove the epidemiological validity.

## Introduction

Highlights

- Contrast-induced nephropathy is one of the major complications of using contrast media, especially in the setting of acute coronary syndrome, and is associated with increased risk of mortality and morbidity.
- It is possible that renal damage starts immediately after using contrast media and evidence shows that compared to serum creatinine levels, gamma-glutamyl transferase levels increase earlier.
- Higher levels of serum gamma-glutamyl transferase appears to be significantly associated with the occurrence of contracted induced nephropathy after coronary artery catheterization and could be considered as a beneficial predictor in the development of contrast-induced nephropathy after cardiac catheterization procedures.

Ischaemic heart disease, which accounts for 16% of all fatalities worldwide, is one of the diseases with a high mortality rate. It is among the main causes of disability-adjusted life-years in age groups 50 and older^1,2^. Acute coronary syndrome (ACS), stable angina, and silent myocardial ischaemia are three different kinds of ischaemic heart disease^3^. Unstable angina, non-ST-segment elevation myocardial infarction, and ST-segment elevation myocardial infarction are all examples of ACS, which is nearly always symptomatic and is caused by a rapid decrease in myocardial blood supply^3,4^.

The electrocardiogram results, the patient’s clinical symptoms, and the biochemical proof of myocardial infarction all contribute to the diagnosis of ACS. Invasive coronary angiography (CAG) is, nevertheless, regarded as the gold standard for identifying coronary artery stenosis^5,6^.

The treatment of ACS consists of pharmacologic therapies and invasive strategies, including CAG and percutaneous coronary intervention (PCI), which are the most effective treatment methods^7^. However, these treatments are accompanied by complications that can be life life-threatening, like contrast-induced nephropathy (CIN)^8^.

CIN, which is also called contrast-induced acute kidney injury (AKI), is defined as AKI occurring after radiographic contrast media (CM) exposure without an alternative aetiology^9^. It is the third most common reason for hospital-acquired acute renal damage, which increases the risk of morbidity and death as well as lengthens of hospitalizations^10,11^.

It is still unclear exactly how CIN’s pathophysiology works. However, it might be accounted for by the intricate interplay of multiple processes, such as vasoconstriction, oxidative stress, renal medullary ischaemia, and the direct toxic effects of CM on the tubules^12,13^.

Serum creatinine (sCr) level is used to make the diagnosis of CIN since it typically rises within 24–48 h and peaks three to five days after CM exposure^14^. Renal injury may begin right away after taking CM, and the rise of sCr happens when more than half of the nephrons are damaged. The rise of the sCr level also depends on different variables including age, sex, weight, muscle mass, medication consumption, and hydration state^15,16^. The aforementioned variables make sCr a delayed and sporadic indicator that may underestimate the prevalence of CIN. Therefore, developing new laboratory biomarkers to anticipate the emergence of CIN is crucial.

A number of studies have found various serum or urinary indicators for predicting CIN, including N-Acetyl-β-glucosaminidase, neutrophil gelatinase-associated lipocalin, interleukin 18, L-fatty acid binding protein, retinol-binding protein, cystatin C, midkine, (NAG) kidney injury molecule-1, and β2-microglobulin.

Gamma-glutamyl transferase (GGT) is a plasma membrane-bound enzyme that facilitates the transmembrane transfer of glutathione, a component essential to intracellular antioxidant processes, and it has been shown that its activity rises in response to oxidative stress^17,18^. GGT has been linked to the development of CIN in certain investigations, but the findings are debatable^19–22^. To ascertain the prognostic efficacy of GGT for CIN in patients with cardiac catheterization, we conducted this systematic review and meta-analysis.

## Methods

The current study examines the predictive value of GGT in CIN through a systematic review and meta-analysis. This study was designed and conducted in 2021–2022. The Preferred Reporting Items for Systematic Reviews and Meta-Analyses (PRISMA) guideline was used to report the study’s findings (Table S1)^23^, Supplemental Digital Content 1, http://links.lww.com/MS9/A163. Additionally, the current investigation was carried out using the AMSTAR 2 checklist (Table S2), Supplemental Digital Content 1, http://links.lww.com/MS9/A163.

### Search strategy

We carried out a thorough search of the literature in three databases (PubMed, Scopus, and Web of Science) for relevant research released between inception and November 2022. We used Google Scholar to search for grey literature. We also looked through the included studies’ reference lists. The following keywords were utilized: contrast media, contrast agent, contrast materials, radiocontrast media, radiocontrast agent, radiopaque media, AKI, acute renal injury, acute renal insufficiency, acute kidney failure, renal failure, contrast nephropathy, CIN, GGT, GGTP, glutamyl transpeptidase or glutamyl transferase, liver function test, and inflammatory markers. The search process was done by two investigators.

### Eligibility criteria

The following criteria had to be fulfilled by all studies to be included: 1—Serum GGT level was investigated as a biomarker to predict CIN in patients undergoing PCI or CAG. 2—CIN was characterized as a 0.5 mg/dl or 25% rise in serum creatinine levels compared within 72 h of the contrast agent’s administration, to the pre-contrast assessments. 3—Original articles with adult patients. 4—papers were published in English. Studies meeting the following criteria were excluded: Irrelevant article types like animal studies, letters, editorials, guidelines, case reports, replies, conference abstracts, clinical trials, reviews, or meta-analyses.

### Data collection and data analysis

Two reviewers independently extracted data from each eligible study using a standardized Excel sheet for data extraction. To check for inconsistencies, a third reviewer evaluated the extracted data. the following information was taken from the included studies: Study characteristics (first author’s name, publication date, study design, and country), patient characteristics (numbers of patients, sex distribution, and mean age), diagnostic criteria of CIN, and outcomes. We contacted the corresponding author for the necessary information.

### Quality assessment

To assess the potential of bias across the studies, we employed the Joanna Briggs Institute Critical Appraisal Checklist^24^. There are 11 items on this scale that evaluate selection bias, information bias, misclassification bias, confounding control measures, and various other significant methodological concerns. To categorize the methodological quality among studies, low, moderate, and high risks of bias were determined for each study.

### Data synthesis

Comprehensive meta-analysis version 3 was used to analyze the findings. The threshold for significance was set at 0.05. We used the Cochran test and I^2^ statistics to assess the degree of study heterogeneity. For heterogenic studies (I^2^>50%, *P*<0.1), we employed a random effect model; otherwise, a fixed effect was utilized. We carried out a sensitivity analysis to assess the impact of each individual study on the overall pooled effect. To determine the number of participants required to reject the null hypothesis, a power analysis was done. Using Grading of Recommendations Assessment, Development, and Evaluation (GRADE) profiler version 3.6 and the GRADE criteria, we assessed the epidemiologic strength of the findings^25^. In order to assess publication bias, we employed Egger’s regression test.

## Results

### Study selection

**Figure 1:**
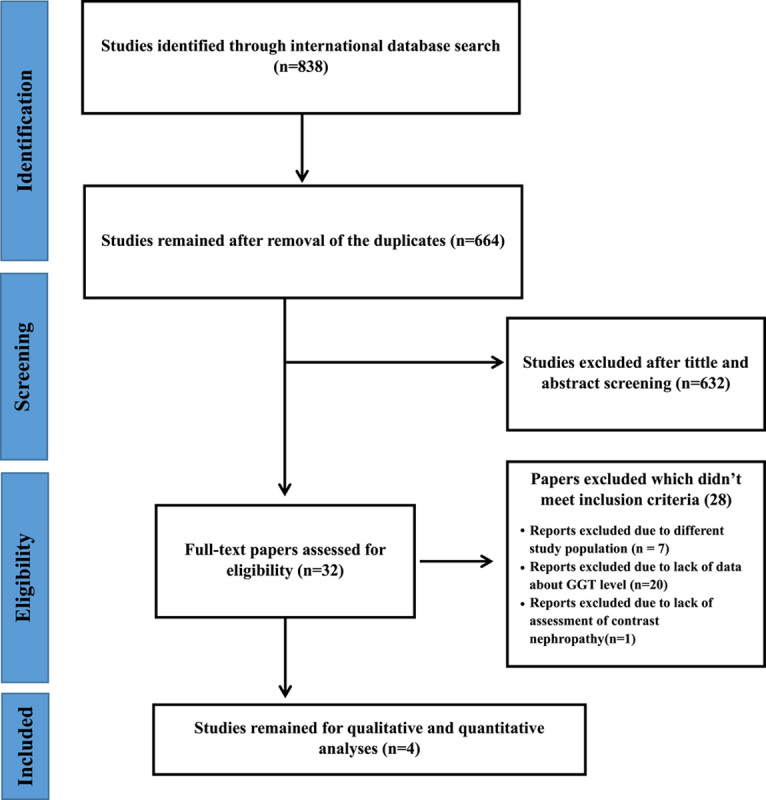
Flowchart of the included studies in the systematic review. GGT, gamma-glutamyl transferase.

After the initial search of PubMed, Scopus, and Web of Science databases, a total number of 838 studies were identified, which 174 studies were duplicated. By screening the title and the abstract of 664 remaining articles 632 studies got excluded. We assessed the full text of 32 studies based on our objectives, and finally, a total number of four studies were included in the final analysis (Fig. 1).

### Study characteristics

Our investigation included a total of four studies with 1346 patients (385 females and 961 males. One study was a prospective cohort, three studies were retrospective cohorts. In all investigations, CIN was considered as a rise in serum creatinine levels of 0.5 mg/dl or 25% from baseline within 72 h of CM injection. Table 1 lists the features of the collected articles in summary form.

**Table 1:** Characteristics of included studies

References	Year	Location	Study design	No. patients	Male, *n* (%)	Study population	CIN incidence%
Yildirim *et al*.^19^	2019	Turkey	Retrospective cohort	200	124 (62)	Patients With ACS undergoing emergency CAG	19.1
Oksuz *et al*.^20^	2015	Turkey	Retrospective cohort	473	367 (78)	Patients With STMI undergoing p-PCI	16.91
Aksoy *et al*.^21^	2020	Turkey	Prospective cohort	300	243 (81)	Patients With STMI undergoing p-PCI	16.7
Toporak^22^	2022	Turkey	Retrospective cohort	373	227 (60.8)	Patients With NSTEMI undergoing CAG	27

ACS, acute coronary syndrome; CAG, coronary angiogram; CIN, contrast-induced nephropathy; NSTEMI, non-ST-elevation myocardial infarction; p-PCI, primary percutaneous coronary intervention; STMI, ST elevation myocardial infarction.

### Risk of bias assessment

We used Joanna Briggs Institute (JBI) Critical Appraisal Checklist for Cohort Studies to evaluate the risk of bias across the studies. For each study, low (quality score below 5), moderate (quality score between 5 and 9), and high risk of bias (quality score above 9) were determined to classify the methodological quality across studies. All included studies were considered to have high quality (Table 2).

**Table 2:** Risk of bias assessment of studies based on Joanna Briggs Institute Critical Appraisal Checklist

References	Q1	Q2	Q3	Q4	Q5	Q6	Q7	Q8	Q9	Q10	Q11	Total score
Yildirim *et al*.^19^	Yes	Yes	Yes	Yes	Yes	Yes	Yes	Yes	Yes	Yes	Yes	11
Oksuz *et al*.^20^	Yes	Yes	Yes	Yes	Yes	Yes	Yes	Yes	Yes	No	No	9
Aksoy *et al*.^21^	Yes	Yes	Yes	Yes	Yes	Yes	Yes	Yes	Yes	Yes	No	10
Toporak^22^	Yes	Yes	Yes	Yes	Yes	Yes	Yes	Yes	Yes	Yes	No	10

JBI checklist questions for cohort studies:

1. Were the two groups similar and recruited from the same population? 2. Were the exposures measured similarly to assign people to both exposed and unexposed groups? 3. Was the exposure measured in a valid and reliable way? 4.Were confounding factors identified? 5. Were strategies to deal with confounding factors stated? 6. Were the groups/participants free of the outcome at the start of the study (or at the moment of exposure)? 7. Were the outcomes measured in a valid and reliable way? 8. Was the follow up time reported and sufficient to be long enough for outcomes to occur? 9. Was follow up complete, and if not, were the reasons to loss to follow up described and explored? 10. Were strategies to address incomplete follow up utilized? 11. Was appropriate statistical analysis used?

### Results of meta-analysis

**Figure 2:**
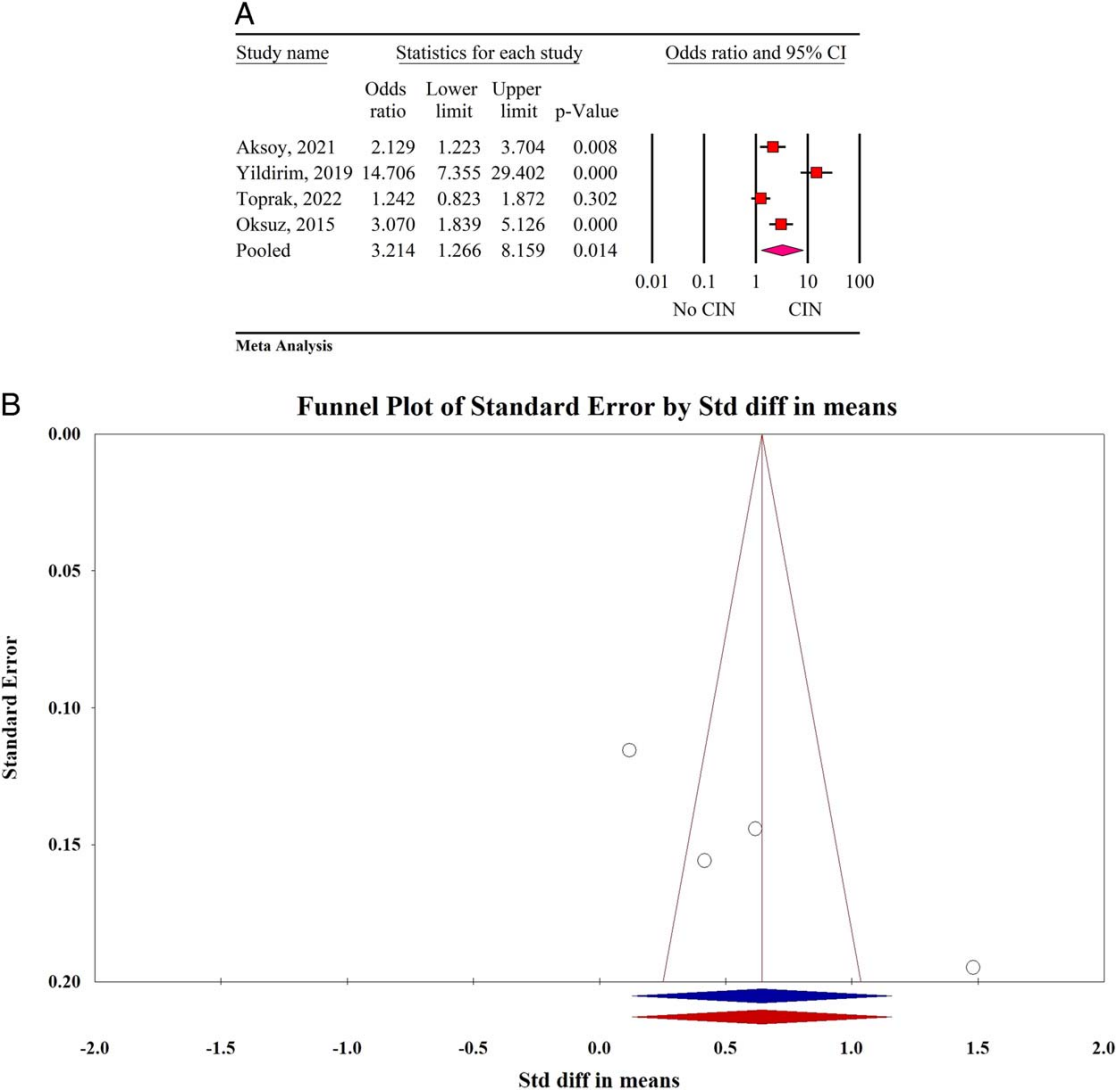
(A) Forest plot for the relationship between GGT and CAG/PCI-induced CIN. (B) The result of publication bias. CAG, coronary angiography; CIN, contrast-induced nephropathy; GGT, gamma-glutamyl transferase; PCI, percutaneous coronary intervention.

**Figure 3:**
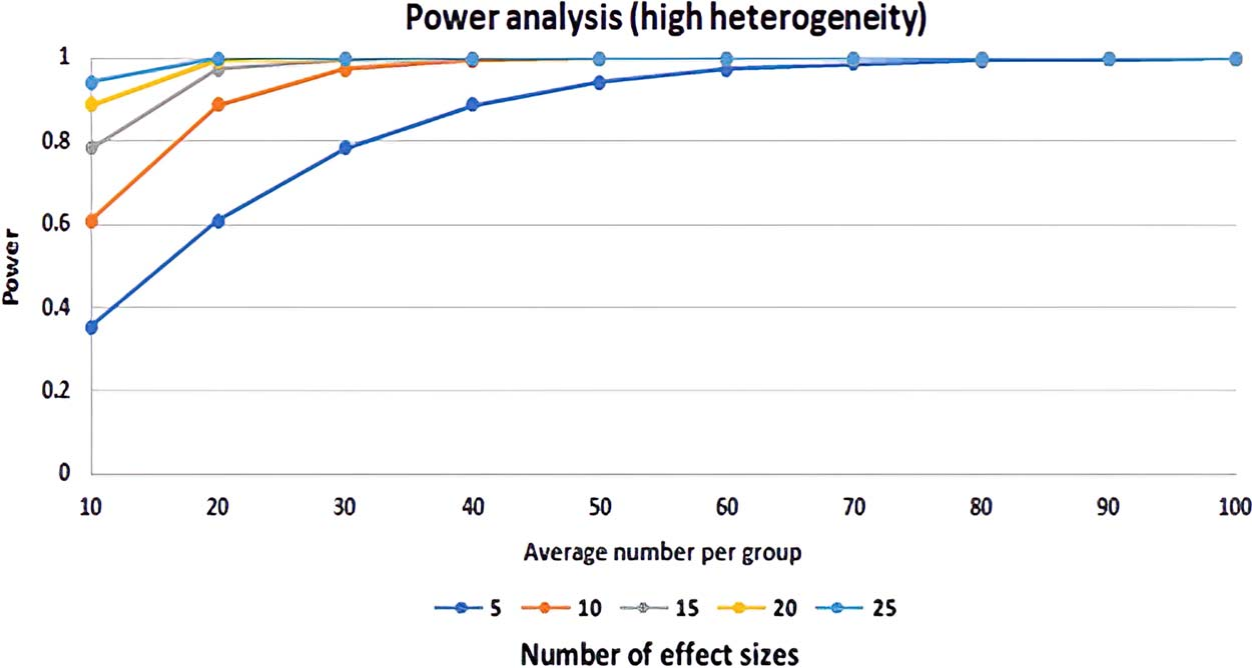
Power analysis for the relationship between GGT and CAG/PCI-induced CIN. CAG, coronary angiography; CIN, contrast-induced nephropathy; GGT, gamma-glutamyl transferase; PCI, percutaneous coronary intervention.

Based on the results of the meta-analysis, higher level of serum GGT was significantly associated with the occurrence of CIN after CAG or PCI (odds ratio: 3.21, 95% CI: 1.26–8.15, *P*=0.014, Fig. 2A). The result of Egger’s regression test showed no significant publication bias (*P*=0.06) (Fig. 2B). The sensitivity analysis result showed significant change in pooled effect size after the removal of Yildirim, 2019^19^ (odds ratio: 1.97, 95% CI: 1.13–3.42, *P*=0.014). Cochran test and I^2^ statistics showed high heterogeneity of the result (Q value: 37.20, I^2^=91.93%, *P*<0.001). The result of sensitivity analysis revealed that by removal of Yildirim and colleagues heterogeneity decreases from 91.93 to 73.73%. The result of power analysis showed high power of the final effect size (1-β =1, number of effect sizes=4, the average number per group=336, Fig. 3).

### Certainty of evidence

For assessing the strength of epidemiological evidence, we used GRADE scoring by GRADE Profiler software version 3.6. We scored the included studies based on their risk of bias, inconsistency, indirectness, imprecision, publication bias, and large effect size. The Certainty of evidence was very low, which was attributed to high unexplained heterogeneity (Table 3).

**Table 3:** GRADE scoring of the relationship between GGT and CAG/PCI-induced CIN

Quality assessment							No. patients		Effect		
No. studies	Design	Risk of bias	Inconsistency	Indirectness	Imprecision	Other considerations	Increased serum GGT	Control	Relative (95% CI)	Absolute	Quality
4	Observational studies	No serious risk of bias	Very serious^a^	No serious indirectness	No serious imprecision	Strong association^b^	268	1078	—	SMD: 0.64 (0.13–1.15 )	Very low

a High unexplained heterogeneity was observed among the included studies.

b The pooled odds ratio is higher than 2.

CAG, coronary angiography; CIN, contrast-induced nephropathy; GGT, gamma-glutamyl transferase; GRADE, Grading of Recommendations Assessment, Development, and Evaluation; PCI, percutaneous coronary intervention.

## Discussion

To the best of our knowledge, so far, our study is the first meta-analysis review that suggests GGT level as a useful biomarker to predict CIN in patients with ACS undergoing cardiac catheterization. GGT, which has the ability to transfer amino acids across cell membranes, is found in serum as well as in many organs such as the liver, pancreas, gut, lungs, and kidneys^26^. In the kidneys, GGT is mainly found in the proximal tubule and Henle loop cells. Along with alanine and aspartate aminotransferases, the GGT level is employed as a marker for the functioning of the liver. However, multiple studies indicate that GGT level also serves as a long-term indicator of renal function and microalbuminuria^27,28^. GGT is released into the blood and urine during AKI when renal tubular cells are destroyed. The level of serum GGT may also serve as an indicator of oxidative stress. Moreover, it may be a biomarker for the cardiometabolic process, according to several studies. Serum GGT levels have been observed to significantly and independently correlate with both cardiovascular and all-cause mortality^29–32^.

CIN is the major complication after using CM, especially in the setting of ACS, and is associated with increased risk of mortality and morbidity^10,33^. As a result, it’s critical to identify patients who could have CIN. The current diagnostic method for CIN relies on variations in serum creatinine levels before and after the injection of CM. A number of variables, such as age, sex, weight, muscle mass, medications, and level of hydration, can affect serum creatinine levels, which rise when more than half of the nephrons are damaged. Renal injury may start happening right once after using CM^15,16^. Compared to serum creatinine levels, GGT levels increase earlier^34^. Usually, serum creatinine level increase in 24–48 h after administration of contrast medium and peak at 3–5 days; hence creatinine level is not the ideal marker of renal injury^14,35^.

In the present meta-analysis, we found that GGT is significantly associated with CIN after cardiac catheterization; however, our results were accompanied with significant heterogeneity and very low certainty of evidence, hence the results of our study may be changed by further future investigations. Low number of included studies and total sample size are the possible justifications of heterogeneity in our study.

**Figure 4:**
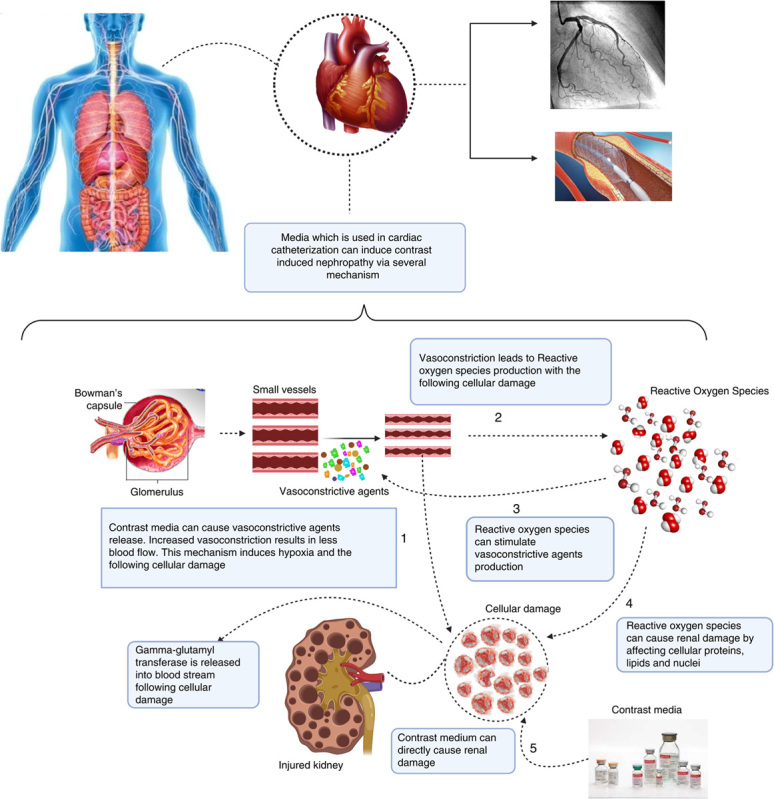
Mechanism of the relationship between GGT and CAG/PCI-induced CIN. CAG, coronary angiography; CIN, contrast-induced nephropathy; GGT, gamma-glutamyl transferase; PCI, percutaneous coronary intervention.

Although the pathogenesis of CIN is not entirely clarified, some mechanisms have parts in the pathogenesis of CIN, including direct nephrotoxic effects of contrast agents, hemodynamic changes, oxidative stress, tubular toxicity, and epigenetic regulations^36^. Some mechanisms are discussed in detail as below:

1—Hemodynamic changes (Fig. 4): Previous studies demonstrated a biphasic hemodynamic response to CM injection. A transient initial increase of renal blood followed by a prolonged decrease of 10–25% less than baseline^37–39^. The latter response phase is a result of vasoconstrictor agents (endothelin, angiotensin-II and vasopressin) dominancy to vasodilator agents (adenosine, nitric oxide, prostaglandin E2, and dopamine) that happens after CM administration^40^. The renal blood flow reduction leads to hypoxia and cellular damage.

2—Oxidative stress (Fig. 4): renal cellular hypoxia can promote reactive oxygen species (ROS) production that results in the dominancy of oxidative stress to antioxidant activities. ROS can directly damage renal cells by affecting cellular lipids, proteins, and nuclei. ROS can also increase vasoconstriction by increasing angiotensin-II and endothelin-I and decreasing nitric oxide, which ultimately results in hypoxia^41^.

3—Tubular toxicity (Fig. 4): CM can directly affect renal cells. It can cause renal cell damage by increasing intracellular ROS levels and triggering apoptotic activities^42^. Other direct effects of CM include intercellular junctions’ disruption, cell proliferation reduction, lowering extracellular calcium, and mitochondrial activity alterations^40,42^.

Among all possible CIN mechanisms, reactive oxygen species significantly promote CIN. Oxidative stress can increase the levels of reactive oxygen species^43^. Different mechanisms were considered to define the association between GGT level and CIN. Serum GGT level could be regarded as a marker of oxidative stress so that it could be associated with the production of ROS^32^. Previous studies showed increased urinary GGT after CM administration^44^. As we determined in this meta-analysis, serum GGT level could be used as an independent factor in predicting CIN. Adding serum GGT level to the current risk score models, such as the validated Mehran risk score, may remarkably improve CIN prediction^45^.

### Limitations

Our study has the following limitations. First, all studies considered in this meta-analysis were single-centre studies that only included Turkish, and the total sample size was relatively small. Due to the homogeneity of the population, the results may be affected by genetic factors. Secondly, the exact cutoff of GGT is not determined in this study because of lack of data. Thirdly, in addition to the GGT level alterations, CIN may be affected by various risk factors, leading to inaccurate results. However, because of the multivariable adjustment considered in the included studies, the relationship between GGT level and CIN is independent and convincing.

## Conclusion

In conclusion, our findings suggest that GGT level could be considered as a beneficial indicator to predict CIN in patients with ACS who are undergoing cardiac catheterization. Increasing serum GGT levels in these patients is an alarm sign and should be considered in the managements of patients who are candidates of cardiac catheterizations.

## Ethical committee approval

This systematic review and meta-analysis do not require an ethical approval.

## Consent

None.

## Source of funding

NA.

## Authors contribution:

Conceptualization: S.H. and E.A.S. Data curation: M.-H.K and M.J. Formal analysis: S.H., and E.A.S. Methodology: M.-H.K., M.J., A.M. Writing—original draft: M.H.K., P.P., M.R.J. Editing: all authors.

## Conflicts of interest disclosure

The authors declare that they have no financial conflict of interest with regard to the content of this report.

## Research registration unique identifying number (UIN)

None.

## Guarantor

Soheil Hassanipour.

## Data availability

The datasets used and/or analyzed during the current study are accessible from the corresponding author on reasonable request.

## Provenance and peer review

Not commissioned, externally peer-reviewed.

## Supplementary Material

## Acknowledgements

The authors acknowledge BioRender since all the illustrations are created with BioRender.com.
